# Neuroprotective Effect of Erythropoietin against Pressure Ulcer in a Mouse Model of Small Fiber Neuropathy

**DOI:** 10.1371/journal.pone.0113454

**Published:** 2014-11-25

**Authors:** Aurore Danigo, Laurent Magy, Laurence Richard, Alexis Desmoulière, Sylvie Bourthoumieu, Benoît Funalot, Claire Demiot

**Affiliations:** 1 Université de Limoges, 3503 GEIST (Institut Génomique Environnement Immunité Santé et Thérapeutique), EA (Equipe d'accueil) 6309 “Maintenance myélinique et neuropathies périphériques,” Faculté de Médecine et de Pharmacie, Limoges, France; 2 CHU (Centre Hospitalier Universitaire) de Limoges, Service de Neurologie, Centre de référence national « Neuropathies périphériques rares », Limoges, France; Rutgers University, United States of America

## Abstract

An increased risk of skin pressure ulcers (PUs) is common in patients with sensory neuropathies, including those caused by diabetes mellitus. Recombinant human erythropoietin (rhEPO) has been shown to protect the skin against PUs developed in animal models of long-term diabetes. The aim of this work was to determine whether rhEPO could prevent PU formation in a mouse model of drug-inducedSFN. Functional SFN was induced by systemic injection of resiniferatoxin (RTX, 50 µg/kg, i.p.). RhEPO (3000 UI/kg, i.p.) was given the day before RTX injection and then every other day. Seven days after RTX administration, PUs were induced by applying two magnetic plates on the dorsal skin. RTX-treated mice expressed thermal and mechanical hypoalgesia and showed calcitonin gene-related peptide (CGRP) and substance P (SP) depletion without nerve degeneration or vascular dysfunction. RTX mice developed significantly larger stage 2 PUs than Vehicle mice. RhEPO prevented thermal and mechanical hypoalgesia and neuropeptide depletion in small nerve fibers. RhEPO increased hematocrit and altered endothelium-dependent vasodilatation without any effect on PU formation in Vehicle mice. The characteristics of PUs in RTX mice treated with rhEPO and Vehicle mice were found similar. In conclusion, RTX appeared to increased PU development through depletion of CGRP and SP in small nerve fibers, whereas systemic rhEPO treatment had beneficial effect on peptidergic nerve fibers and restored skin protective capacities against ischemic pressure. Our findings support the evaluation of rhEPO and/or its non-hematopoietic analogs in preventing to prevent PUs in patients with SFN.

## Introduction

Prolonged pressure and the resulting local ischemia are widely accepted as the primary etiologies of skin pressure ulcers (PUs) [1], [2], but the precise mechanisms of their formation remain unclear. PUs develop when an extended period of uninterrupted pressure on one part of the body results in reduced blood supply causing deficient tissue nutrition and ischemia [3]. The increased risk of PUs observed in patients affected by sensory neuropathy is generally attributed to the sensory loss, which allows prolonged painless pressure at compression point [4]. In order to further study the link between sensory nerve dysfunction and PUs, we developed a purely functional and reversible mouse model of small fiber neuropathy (SFN) induced by resiniferatoxin (RTX) [5]. RTX is an ultrapotent capsaicin analog that acts on transient receptor potential vanilloid 1 (TRPV1). TRPV1 is a cation channel expressed by both sensory Aδ- and C-fibers [6]. Several studies have shown that TRPV1-expressing sensory nerves are involved in tissue protection during ischemia in the heart or limbs [7]–[11]. Overstimulation of TRPV1 by RTX induces neuropathies affecting only small nerve fibers and characterized by neuropeptide depletion and/or by nerve degeneration in rodents [12]–[14]. The neurotoxicity of vanilloids may differ dramatically depending on doses, administration modalities, animal species and method by which unmyelinated fibers are visualized [12]. In our model, administration of RTX in mice induced classical mechanical and thermal hypoalgesia. However, RTX-induced hypoalgesia did not result from nerve degeneration but was associated with depletion of calcitonin gene-related peptide (CGRP) and substance P (SP) in peptidergic nerve fibers [5]. Peptidergic nerve fibers have been reported to have ischemic-protective properties [10], [15], but have not been assessed for PU development, which combines pressure/loading and local ischemia. Stimulation of these nerve fibers mediates antidromic transport and CGRP/SP release by peripheral nerve endings; in addition, CGRP and SP are key players in skin homeostasis and have protective effects on ischemic injury in various tissues [16]–[18]. We then hypothesized that CGRP and SP, released by epidermal nerve endings could be involved in cutaneous protection against PU formation.

The presence of EPO and its receptor in peripheral axons and DRG neurons suggests a role in neuronal functions [19]. Erythropoietin is well known for its neuroprotective and neurorepairing effects [20]. For example, in chemotherapy-induced neuropathy models, recombinant human erythropoietin (rhEPO) improves nociceptive behaviors and sensory nerve conduction, protects against axonal degeneration, without impairing anti-tumor activity [21]–[24]. In diabetic animal models, rhEPO reduces functional symptoms, axonal dysfunction and increases IENFs density [25]–[27]. In a previous study, recombinant human erythropoietin (rhEPO) was shown to prevent stage 2 PU formation and to impede degeneration of cutaneous sensory nerves in a long-term mouse experimental diabetes [26]. However, hyperglycemia induces oxidative stress, vascular and inflammatory disorders which may contribute to diabetic neuropathy, and other outcomes. The neuroprotective effect of rhEPO in the diabetic mouse model could be masked by its endothelial, anti-inflammatory and anti-oxidant properties [28]. Moreover, diabetic neuropathy impairs sensory, autonomic and motor nerves. Thus, the neuroprotective effect of rhEPO might not be limited to diabetes-induced neuropathy. The effects of rhEPO on small nerve fiber dysfunction and ischemic skin injury have never been studied in non-diabetic animal models. Thus, the aims of the present study were to determine (i) whether rhEPO could prevent RTX-induced SFN, (ii) whether peptidergic TRPV1-expressing cutaneous nerve fibers, could be involved in skin protection against PU formation, and (iii) whether rhEPO could prevent PU formation by improving small nerve fiber function.

## Methods

The study was carried out according to the guidelines for ethical care of experimental animals of the European Community and was approved by the French Agriculture Ministry (authorization n°87-019). The protocol was approved by the Ethics Committee of Animal Experiments of Limousin (Comité Régional d'Ethique pour l'Expérimentation Animale, CREEAL. Permit numbers: 1-2013-1, 2-2013-2). According to the experiments, animals were anesthetized by intraperitoneal injection of thiopental sodium or by isoflurane inhalation. For tissue collection, the animals were sedated by isoflurane, and immediately euthanatized by cervical dislocation. Every effort was made to minimize suffering and numbers of animals used in the following experiments.

### 1. Animals and treatments

**Figure 1:**
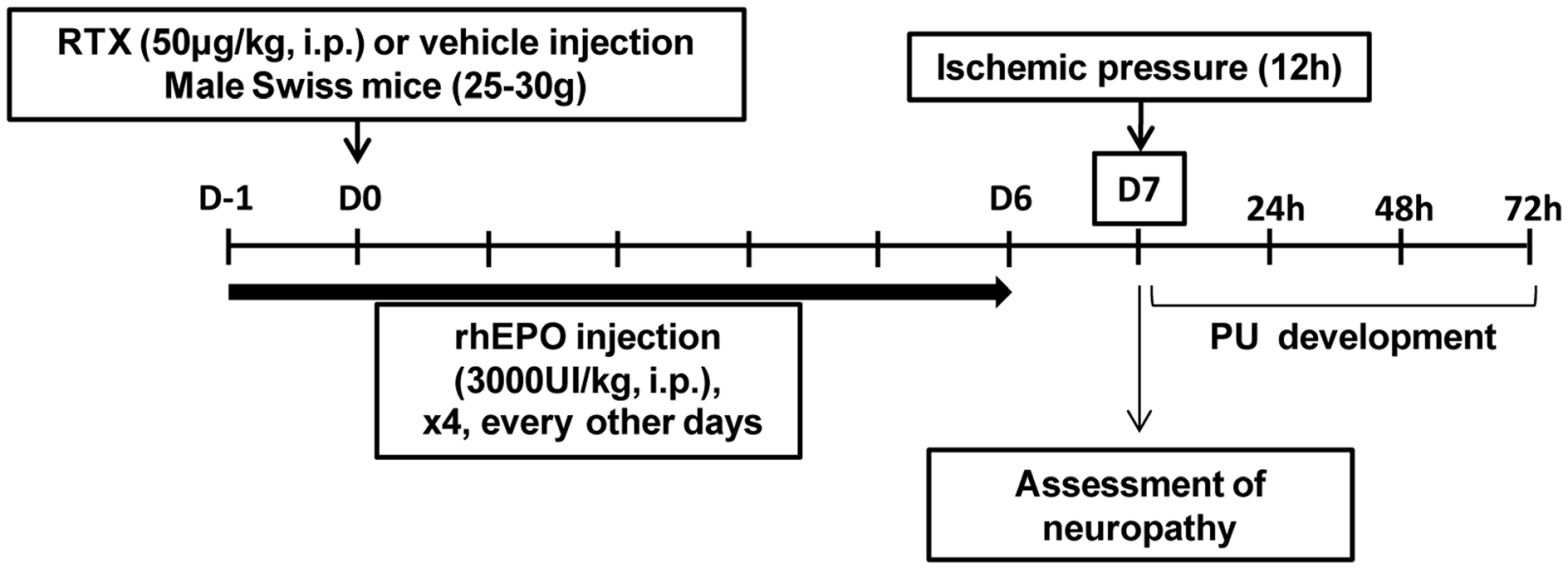
Schematic representation of study design.

Experiments were performed on young male Swiss mice (20–25 g) from Centre d'Elevage Depré. The mice were randomly assigned to four weight-matched groups: Vehicle, RTX, RhEPO and RTX-rhEPO. Animals were housed in plastic cages and maintained on a 12 h light/dark cycle with food and water *ad libitum*. The animals were allowed to adapt to this environment for a period of 7 days before the experiments. SFN was induced by a single injection of RTX (50 µg/kg, i.p. Sigma-Aldrich, Lyon, France) and Vehicle mice received an equivalent volume of vehicle (10% DMSO, i.p.). RhEPO treatment consisted of four injections (3000 UI/kg, i.p.); one 24 h before RTX or vehicle injection and every other days after RTX or vehicle injection during 6 days [26]. Untreated mice received four injections of an equivalent volume of saline solution. Experiments were performed seven days after RTX/vehicle injection (Figure 1). Hematocrit was determined by capillary centrifugation (10 000 rpm, 15 min) from blood samples of submandibular bleeding seven days after RTX/vehicle injection. To assess the relationship between neuropeptide depletion and PU, the mice received injections of CGRP antagonist CGRP 8-37 (200 nmol/kg, i.p, Tocris Bioscience), NK_1_ antagonist SR140333 (148 nmol/kg, i.p., Tocris Biscience) or Saline (0.1% ethanol in saline solution) every 12 h from the time of magnet application.

### 2. Evaluation of nociceptive behaviors

#### 2.1 Hot-plate test

Thermal withdrawal latencies were measured with a 52°C hot-plate (Bioseb, France). Each test session consisted of three trials separated by 15 min. To assess the thermal withdrawal latencies, nociceptive behaviors, like jumping or hind paw shaking and licking, were looked for. The cutoff limit was 25 s to avoid potential tissue damage. The mean latency was expressed as the threshold of an individual animal to nociceptive thermal stimulation [13], [29].

#### 2.2 Mechanical pressure algesia

Tail pressure thresholds were recorded with the Paw/Tail Pressure Analgesia meter of the Randall-Sellito test (Bioseb, Vitrolles, France). A 16 g.s^−1^ linear increasing pressure increasing was applied to the base of the tail with a cut off at 250 g to avoid tissue injury. The tail pressure at which biting or licking behaviors were observed was recorded and expressed in grams. Three tests separated by at least 15 min were performed and the mean value of these tests was calculated to represent the threshold of an individual animal to mechanical pressure algesia [26].

### 3. Evaluation of skin microvascular reactivity

A depilatory lotion was used to remove the animals' hairs 2 days before skin LDF measurements and iontophoretic delivery. Animals were anesthetized with thiopental sodium (65 mg.kg^−1^ intraperitoneally) and then placed in an incubator (Mediprema, Tours, France) to maintain a stable cutaneous temperature (35.0±0.5°C). Non-invasive blood pressure (Bionic Instruments, Tokyo, Japan) was recorded before and after experiments to verify SABP stability.

Skin blood flow was recorded, using a laser Doppler multifiber probe (481-1, Perimed) during transcutaneous iontophoresis applied to a 1.2 cm^2^ area on the hairless back of animals. This method was described by Demiot *et al.*, 2006 [30]. Endothelium-independent response was assessed, using a cathodal sodium nitroprusside (SNP) iontophoretic delivery (67 mmol.L^−1^ Nitriate; SERB, Paris, France) with a current application of 100 µA for 20 s. Endothelium-dependent response was assessed using an anodal acetylcholine (Ach) iontophoretic delivery (5.5 mmol.L^−1^; Sigma, St-Quentin Fallavier, France) with a current application of 100 µA for 10 s. Skin blood flow baselines were recorded before endothelium-dependent and -independent response assessments. Vasodilator responses were reported as the maximal percent increase from baseline in response to iontophoretic SNP or ACh delivery.

### 4. Assessment of neuropathy

#### 4.1 Tissue collection

Sciatic nerves, dorsal root ganglia and footpad skin were removed and fixed for assessment of RTX and rhEPO effect on innervation by immunofluorescence histochemistry and electron microscopy.

#### 4.2 Assessment of footpad skin innervation

Footpad skin was removed with a punch biopsy (Ø 3 mm), fixed 6 h in 4% paraformaldehyde (PFA), cryoprotected (30% sucrose) and then frozen at −20°C. Sections were cut on a cryostat at 20 µm and were incubated overnight with primary antibodies against protein gene product 9.5 (PGP9.5) (1∶600; UltraClone, Island of Wight, UK), SP (1∶100; Millipore, Molsheim, France) or CGRP (1∶1000; Abcam, Paris, France). Sections were then incubated with appropriate secondary antibodies, Cy3-conjugated (1∶500; Jackson Immunoresearch, Suffolk, UK) or AF488-conjugated (1∶500; Life Technologies, Saint-Aubin, France). Epidermal nerve fibers were counted under 400× magnification (Eclipse 50i, Nikon Instruments), following an established protocol [31]. The length of the dermo-epidermal junction was determined with NIS-Elements BR 2.30 software (Nikon). Density of intraepidermal nerve fibers (IENFs) was defined as the number of epidermal nerves divided by the epidermal length.

#### 4.3 Assessment of DRG neurons

For systematic sampling, two lumbar DRG (L4-L5) *per* mice were collected, fixed in PFA, cryoprotected (30% sucrose), frozen at −20°C and then were cut on a cryostat at 8 µm. Double-labeling with Neuron-specific β–III tubulin (clone TUJ-1, 1∶1000, R&D systems, Lille, France) and with SP or CGRP was performed. Each DRG section was photographed under fluorescence microscope (200×) in a systematic fashion. Immunoreactive DRG neurons were counted and only the area containing neurons was measured with NIS-Elements BR 2.30 software (Nikon). The density of TUJ-1(+) neurons was expressed as neurons/mm^2^. The density of peptidergic neurons was expressed as CGRP(+) or SP(+) neurons/TUJ-1(+) neurons.

#### 4.4 Assessment of unmyelinated nerve fibers in sciatic nerves

Sciatic nerves were fixed in 2.5% glutaraldehyde diluted in Sorensen buffer, dehydrated and embedded in Epon 812 resin (Euromedex, France). Semi-thin sections were stained with toluidin blue. Ultrathin sections were stained with uranyl acetate and lead citrate and observed under an electron microscope (Jeol 1011). Photographs were taken at 25,000× magnification. The unmyelinated fibers enclosed within the basal lamina of single Schwann cell (i.e. a Remak bundle) were counted.

### 5. Pressure ulcer study

#### 5.1 Pressure-induced ulcer model

PUs were created on the dorsal skin as described by Stadler *et al.* [32]. The dorsal hair was shaved. After 24 hours, the skin was gently pulled up and placed between two round ceramic magnetic plates (10 mm diameter and 1 mm thick, with an average weight of 0.5 g and 10,000 Gauss magnetic force). Epidermis, dermis, and subcutaneous tissue layer including *panniculus carnosum* muscle (PCM), were pinched with the magnetic plates during 12 h, inducing PUs (stage ≥2) in healthy mice. This process created a compressive pressure of approximately 2,000 mmHg between the two magnets. A pressure greater than 400 mmHg has been estimated to be necessary to maintain microvascular closure in mouse dorsal skin [33]. We decided to apply a static loading of 2,000 mmHg (or 266 kPa) according to previous studies on human [34].

Each compressed area was photographed, 24 h, 48 h and 72 h after pressure release, using a 3.3 megapixel camera (Photo PC 3100Z; Epson, Nagano, Japan). In preliminary studies (data not shown) with this experimental conditions, we observed that PU areas reached a maximum 3 days after pressure release. PUs were visually assessed according to the standardized ulcer scale [32]. Compressed area and skin ulcer area were delimited using NIS-element BR 2.30 software (Nikon), and skin ulcer area percentage was calculated in the total compressed area.

#### 5.2 Histological analysis

Once mice were euthanized, compressed skin samples were dissected with a margin of normal skin at 24 h and 72 h after pressure release, fixed overnight in a formalin solution, and embedded in paraffin. Sections of 7 µm were stained with Masson's trichrome. Histological examinations were analyzed with an optical microscope (Leica).

### 6. Statistical analysis

Prism version 6.04 (GraphPad Software, Inc.; LaJolla, CA, USA) was used to make graphs and perform statistical tests. All data are presented as mean ± SD. A one-way analysis of variance (ANOVA) was used to evaluate differences among multiple groups, with *p* values determined by Bonferroni's multiple comparisons test with Gaussian distribution. A non-parametric Kruskal-Wallis test and Dunn's multiple comparisons test were used for data which did not follow a Gaussian distribution. Differences were considered to be statistically significant at *p*<0.05.

## Results

Seven days after RTX or vehicle administration, there was no difference in body weight between treated and untreated groups (Table 1). RTX and rhEPO did not change systolic arterial blood pressure (SABP). Hematocrit was significantly increased by rhEPO in Vehicle and RTX mice (Table 1).

**Table 1:** Effect of RTX and rhEPO on body weight, SABP, hematocrit and skin microvascular reactivity.

Groups	Vehicle	RTX	RhEPO	RTX-rhEPO
**Body weight (g)**	30.33±0.33	30.71±0.57	32.00±0.57	30.70±0.45
**SABP (mmHg)**	69.80±2.58	75.95±3.24	63.30±3.51	69.00±2.45
**Hematocrit (%)**	**47.89±7.6**	**49.90±5.8**	**61.76±7** **	**65.12±7.7** **
**SNP (%)**	37.1±17.1	30±12.5	37.6±29.8	32.6±17.1
**Ach (%)**	**28.7±9.7**	**31±11.9**	**16.3±6.56** *	**16.5±6** **

Ach: endothelium-dependent vasodilator response to iontophoretic delivery of acetylcholine, rhEPO: recombinant human erythropoietin, RTX: resiniferatoxin, SABP: systolic arterial blood pressure, SNP: endothelium-independent vasodilator response to iontophoretic delivery of sodium nitroprusside. n = 6 in each group, 1-way ANOVA followed by Bonferroni's *post-hoc* test, *p<0.05, **p<0.01: significance of the difference between rhEPO-treated mouse group and respective untreated mouse group.

### 1. RhEPO alters normal skin microcirculation reactivity in RTX mice

Cutaneous temperature and SABP were stable throughout the experiment (Table 1). Endothelium-independent vasodilator responses to iontophoretic delivery of sodium nitroprusside (SNP) were found similar in untreated RTX and Vehicle mice. RhEPO had no effect on endothelium-independent vasodilation function in RTX and Vehicle mice (Table 1).

Endothelium-dependent responses to iontophoretic delivery of acetylcholine (ACh) were found similar in RTX and Vehicle mice, indicating that endothelial function was not altered by RTX. However, rhEPO altered endothelium-dependent vasodilation in RTX and Vehicle mice (Table 1).

### 2. RhEPO improves nociceptive behaviors altered by RTX

**Figure 2:**
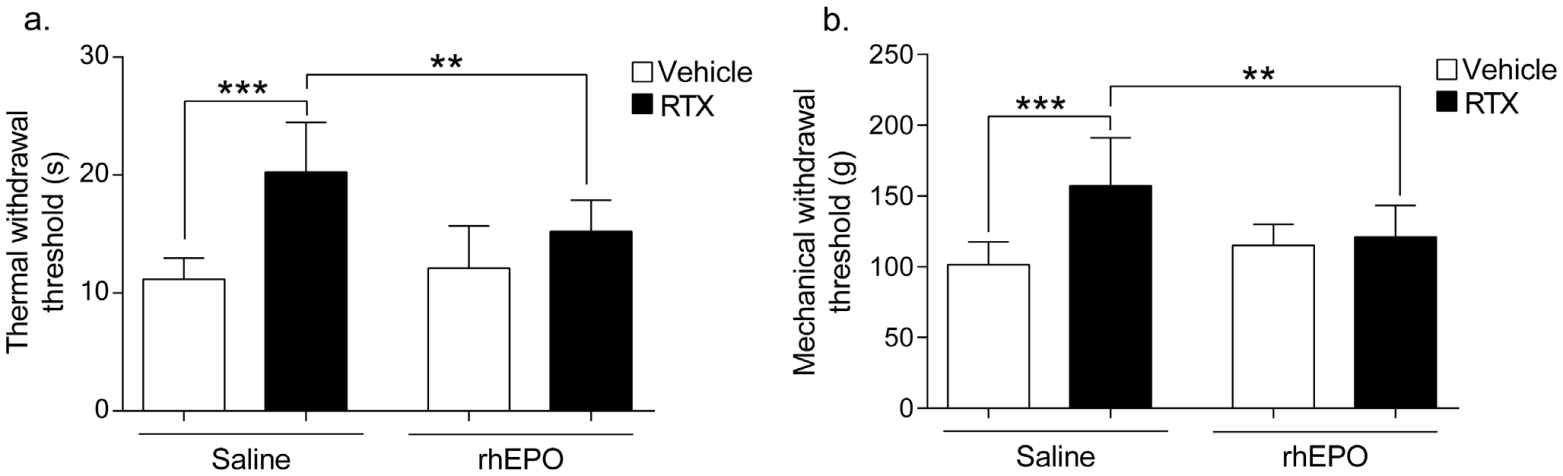
Effects of RTX and rhEPO on thermal and mechanical nociceptive behaviors. (**a**) Hot plate test. Withdrawal latencies to thermal stimuli (52°C). (**b**) Randall-Sellito tail pressure test. Mechanical withdrawal thresholds to nociceptive tail pressure. (n = 10 in each group, 1-way ANOVA followed Bonferroni's *post-hoc* test, **p<0.01, ***p<0.001). rhEPO: recombinant human erythropoietin, RTX: resiniferatoxin.

The plantar thermal nociceptive withdrawal latencies were significantly increased by 81% in RTX compared to Vehicle mice. Withdrawal latencies were unchanged by rhEPO in Vehicle mice (Figure 2a). Thermal nociception was significantly improved in RTX-rhEPO mice compared with RTX mice (Figure 2a). The tail nociceptive pressure threshold was significantly increased by 55% in RTX mice compared to Vehicle mice (Figure 2b). RhEPO had no effect on tail withdrawal latencies in Vehicle mice. RTX mice treated with rhEPO completely recovered mechanical nociception (Figure 2b).

### 3. RhEPO has no effect on unmyelinated fiber morphology and density

**Figure 3:**
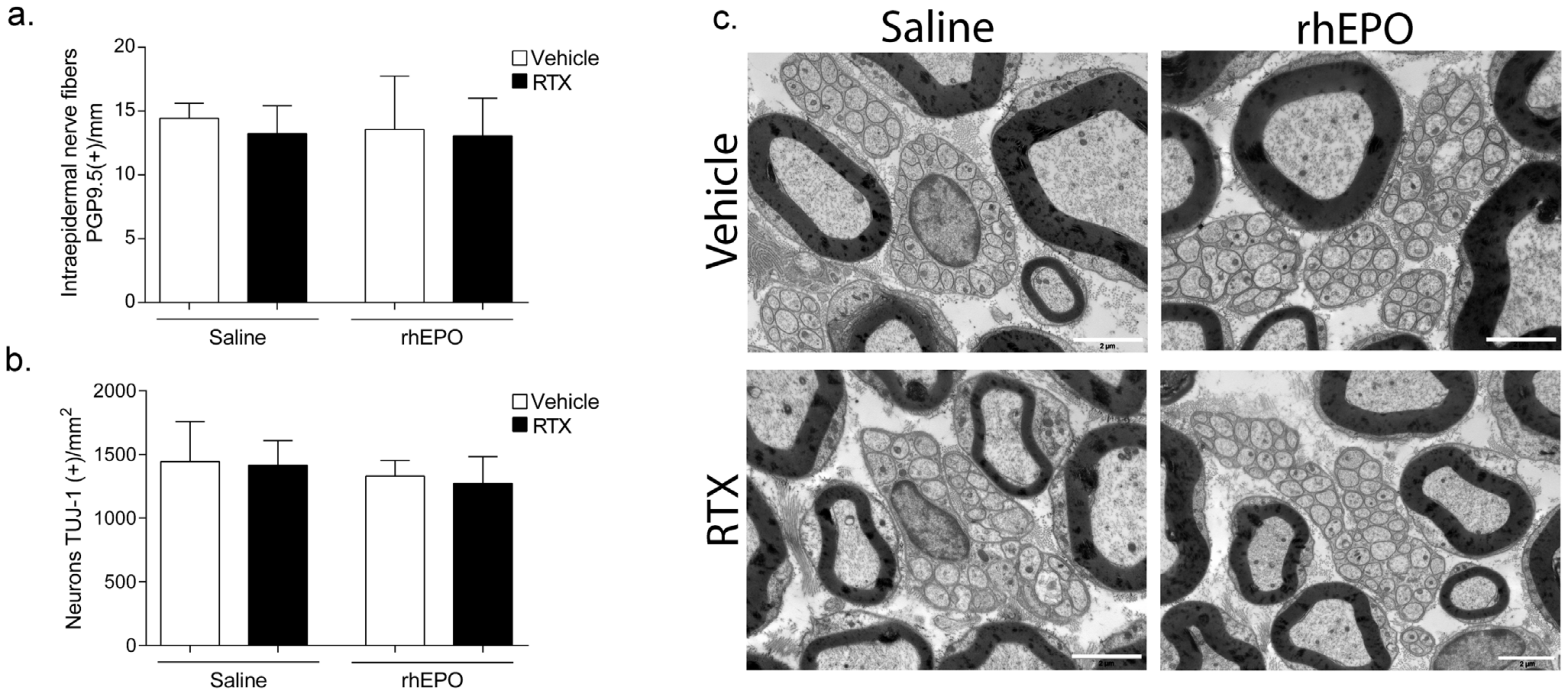
Effects of RTX and rhEPO on unmyelinated nerve fiber and DRG neurons. (**a**) Foot pad skin and DRG neurons were immunostained for protein gene product 9.5 (PGP9.5). Quantification of intraepidermal nerve fiber positive for PGP9.5. The density of intraepidermal nerve fibers was calculated according to ENFS guidelines [33]. (n = 6 in each group). (**b**) Quantification of DRG neurons positive for PGP9.5. The density of neurons was expressed as neurons/square millimeter (n = 12 in each group). (**c**) Unmyelinated nerve fiber morphology in sciatic nerve was examined by electron microscopy. Scale bar = 2 µm. DRG: dorsal root ganglia, rhEPO: recombinant human erythropoietin, RTX: resiniferatoxin.

The density of IENFs was unchanged in RTX mice compared with Vehicle mice (Figure 3a). The density of DRG neurons (β-III tubulin-positive cells) was not affected by RTX injection (Figure 3b). Ultrastructural examination of sciatic nerves confirmed that unmyelinated fiber morphology was undamaged by RTX (Figure 3c). Moreover, the number of unmyelinated nerves enclosed by Schwann cell (Remak bundles) in RTX group (13.7±2.5) was similar to those of the Vehicle group (13.9±2.9, *p*>0.05). RhEPO did not modify unmyelinated fibers density at epidermal and DRG levels (Figure 3a and b). Ultrastructural morphology of unmyelinated and myelinated fibers was undamaged in sciatic nerves of mice treated with rhEPO (Figure 3c). The number of unmyelinated axons per Remak bundle was unchanged by rhEPO treatment (RTX-rhEPO: 14.5±2.7 *vs.* RTX, *p*>0.05. RhEPO: 12.5±0.8 *vs.* Vehicle, *p*>0.05).

### 4. RhEPO reduces CGRP and SP depletion in RTX mice

**Figure 4:**
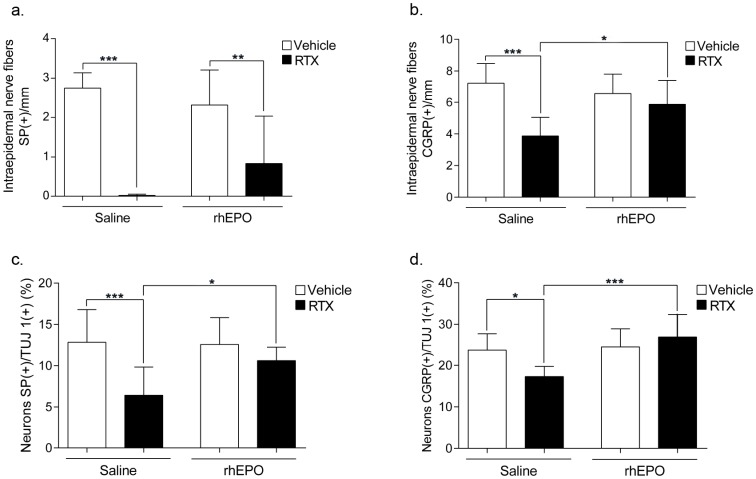
Effects of RTX and rhEPO on IENFs and DRG neurons positive for SP and CGRP. (**a,b**) Footpad skins were immunostained for SP (a) or CGRP (b). The density of IENFs was calculated according to ENFS guidelines [33] (n = 6 in each group). (**c,d**) DRG neurons were double-immunostained for SP/TUJ-1 (c) or CGRP/TUJ-1 (d). The density of neurons is expressed as neurons SP(+) or CGRP(+)/neurons TUJ-1(+). (n = 12 in each group). 1 way-ANOVA followed Bonferroni's *post-hoc* test *p<0.05, **p<0.01, ***p<0.001. CGRP: calcitonin gene-related peptide, DRG: dorsal root ganglia, IENFs: intraepidermal nerve fibers, rhEPO: recombinant human erythropoietin, RTX: resiniferatoxin, SP: substance P.

RTX induced a large SP depletion (Figure 4a) and a mild reduction of CGRP (Figure 4b) in IENFs of footpad skin. A depletion of SP (Figure 4c) and a mild decrease of CGRP were also observed in the DRG neurons of RTX mice when compared with Vehicle mice (Figure 4d). The density of SP(+) IENFs in RTX-rhEPO mice was higher than in RTX mice, but the difference was not significant (Figure 4a). The amount of SP in the DRG neurons of RTX-rhEPO mice was significantly improved compared with RTX mice and was found similar to RhEPO mice (Figure 4c). The number of CGRP(+) IENFs in RTX-rhEPO mice was significantly higher than in RTX mice, and reached the RhEPO group's values (Figure 4b). The same pattern was observed with the DRG neurons, RTX-rhEPO mice showed a restoration of CGRP, with a significantly higher number of CGRP(+) DRG neurons than in RTX mice (Figure 4d).

### 5. C-peptidergic fiber neuropathy induced by RTX increases PUs development. Substance P and CGRP are independently involved in skin protection against injury

**Figure 5:**
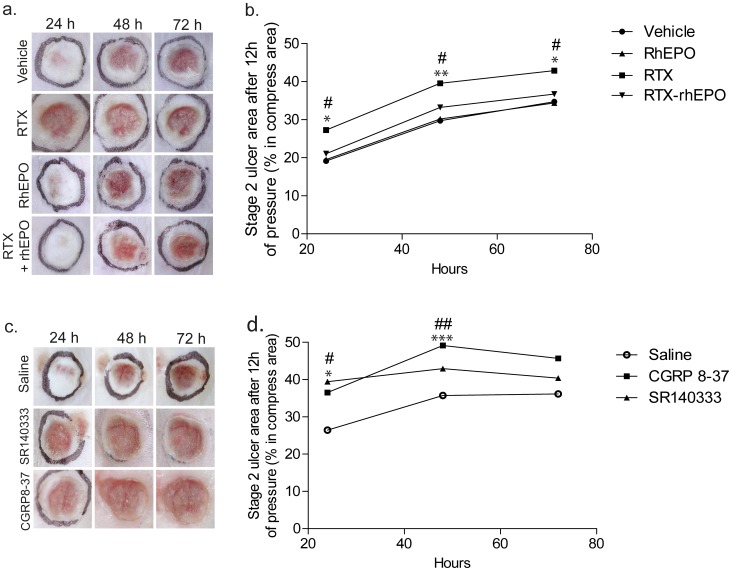
Cutaneous macroscopic findings following 12 h of pressure (**a,b**). Effect of RTX and rhEPO. (a) Macroscopic appearance of pressure-ulcers (PUs) 24 h, 48 h and 72 h after pressure release. (b) Time course of macroscopic stage 2 PU areas. n = 20 in each group, non-parametric Kruskal-Wallis test followed by Dunn's multiple comparisons test *p<0.05, **p<0.01 Vehicle *vs.* RTX, #*p*<0.05 RTX *vs.* RTX-rhEPO. (**c,d**) Effect of SR140333 (NK1 antagonist) and CGRP 8-37 (CGRP antagonist) (c) Macroscopic appearance of pressure-ulcers (PUs) 24 h, 48 h and 72 h after pressure release. (d) Time course of macroscopic stage 2 PU areas. n = 10 in each group,non-parametric Kruskal-Wallis test followed by Dunn's multiple comparisons test, *p<0.05, ***p<0.001, CGRP 8-37 *vs.* Saline mice. #*p*<0.05, SR140333 vs. Saline mice. rhEPO: recombinant human erythropoietin, RTX: resiniferatoxin.

Twenty-four hours after pressure release, the incidence of ischemic lesions in RTX mice was more pronounced than in Vehicle mice; 82% of RTX mice and 55% of Vehicle mice developed a stage 2 PU. Both RTX and Vehicle mice showed a daily progression of PUs' size (PU stage ≥2) from 24 h to 72 h after pressure release with maximum areas at 72 h. Larger PU areas were observed 24 h, 48 h and 72 h after pressure release in RTX compared with Vehicle mice (Figure 5a). Mice treated with SR140333 showed significantly larger stage 2 PUs than saline mice at 24 h and 48 h (Figure 5c and d). Treatment with CGRP 8-37 induced a larger stage 2 PU compared with saline mice, 24 h and 48 h after pressure release. These results suggest that release of SP and CGRP could be crucial to protect skin in early steps of ischemia injury.

**Figure 6:**
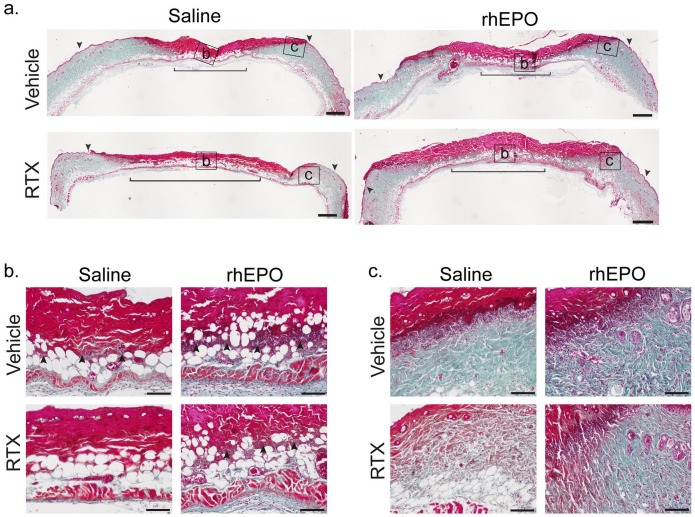
Effects of RTX and rhEPO on cutaneous histological findings 24 h after pressure release. Ischemic skin lesions were removed 24 h after pressure release and stained with Masson's trichrome. (**a**) Histological look of pressure ulcer (PU). Bracket delimits area where necrosis is the most profound and arrows mark the margin of the lesion. Scale bar = 500 µm. (**b, c**) Microphotographs taken from (a) of central compressed area (b) and of ischemic wound margins (c). Arrows indicate infiltrates of inflammatory cells. Scale bar = 100 µm. rhEPO: recombinant human erythropoietin, RTX: resiniferatoxin.

**Figure 7:**
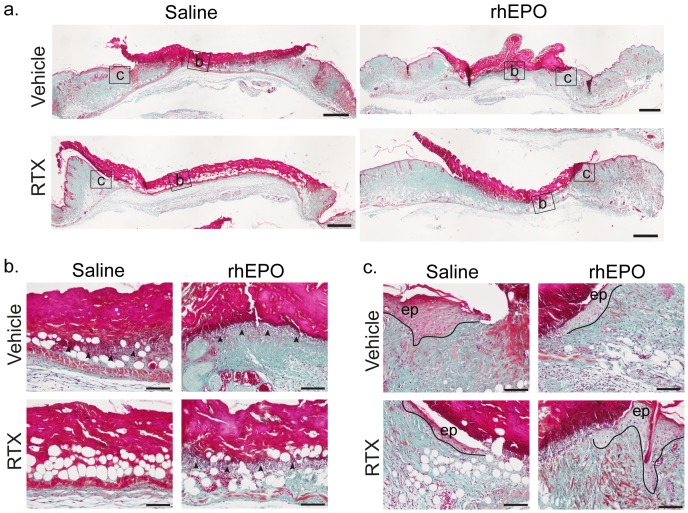
Effects of RTX and rhEPO on cutaneous histological findings 72 h after pressure release. Ischemic skin lesions were removed 72 h after pressure release and stained with Masson's trichrome. (**a**) Histological look of pressure ulcer (PU). Scale bar = 500 µm. (**b, c**) Microphotographs taken from (a) of central compressed area (b) and of ischemic wound margins (c) Arrows indicate infiltrates of inflammatory cells. Scale bar = 100 µm. ep: epidermis, rhEPO: recombinant human erythropoietin, RTX: resiniferatoxin.

Histologically, 24 h after pressure release, necrosis affected epidermis, dermis and subcutaneous layers in the compressed areas of Vehicle and RTX mice, leading to the development of stage 2 PUs (Figure 6). The extent of necrosis affecting all layers of skin was more important in RTX than in Vehicle mice (Figure 6a). In opposition to Vehicle mice, RTX mice did not display an infiltration of inflammatory cells at the center and margins of the ischemic lesion (Figure 6b and c). Skin of untreated mice showed thick bundles of collagen at the ischemic lesion's edges whereas RTX-treated mice's collagen fibers proved to be thin (Figure 6c). Seventy-two hours after pressure release, histology showed a massive infiltration of inflammatory cells in the lesion's center and proliferative epidermis of the wound's margins of Vehicle mice (Figure 7). In contrast, in RTX mice, no inflammatory infiltrates were observed and the epidermis of the wound's margins was visibly thin (Figure 7a, b and c).

### 6. RhEPO prevents the RTX mediated increase in PUs development

Twenty-four hours after pressure release, the incidence of ischemic lesions in RTX-rhEPO mice was less pronounced than in RTX mice; 57% of RTX-rhEPO mice and 82% of RTX mice developed a stage 2 PUs. RhEPO prevented enlargement of PU areas induced by RTX (Figure 5, 6 and 7). Macroscopically, rhEPO had no effect on stage 2 PU formation in Vehicle mice. RTX mice treated with rhEPO showed significantly smaller PU areas than RTX mice 24 h, 48 h and 72 h after pressure release (Figure 5). One day after pressure release, Masson's trichrome staining showed that necrosis affecting epidermis, dermis and subcutaneous tissue was less extensive in RTX-rhEPO mice than in untreated RTX mice (Figure 6a). Collagen fibers at the wound's borders were also found thicker in RTX-rhEPO mice (Figure 6c). In contrast with the RTX group, RTX-rhEPO mice, like both control groups, presented a massive infiltration of inflammatory cells at the ischemic lesion's center and margins (Figure 6b and c). A similar pattern was found three days after pressure release (Figure 7).

## Discussion

The main findings of this report are that (1) Systemic rhEPO treatment prevent RTX-induced neuropathy by its neuroprotective properties (2) A functional SFN induced by RTX with a CGRP and SP depletion, promotes skin PU development, after a long ischemic pressure application (3) PU formation is increased independently by CGRP antagonist (CGRP8-37) and SP antagonist (SR140333) (4) Neuroprotective effect of rhEPO restores skin capacity to protect against ischemic pressure in RTX-induced neuropathy model.

Seven days after intraperitoneal RTX injection, mice revealed a significant thermal and mechanical hypoalgesia. No nerve degeneration in skin, sciatic nerve and DRG was observed. SP was largely depleted in IENFs and DRG neurons, CGRP, though, was only moderately depleted. The main mechanism responsible for DRG neuron degeneration upon exposure to RTX *in vitro* is rapid Ca^2+^ toxicity following TRPV1 activation [35]. *In vivo* and in our condition, systemic RTX administration did not cause nerve degeneration, as shown by light and electron microscopy. Similar findings have been obtained in urinary bladder, where RTX-induced impairment was characterized as a purely functional desensitization without morphological changes of the TRPV1-expressing sensory nerves [36].

In this model, thermal and mechanical latencies were restored by rhEPO treatment which completely prevented SP and CGRP depletion in DRG neurons. RhEPO completely prevented CGRP depletion, and partially averted SP depletion in IENFs. To explain the differences in neuropeptide amounts between DRG neurons and IENFs, we hypothesize that rhEPO either prevented CGRP/SP depletion or stimulated CGRP/SP synthesis [37], in DRG cell bodies, but that neuropeptide transport was incomplete at the skin level. Using this functional SFN model, we showed that rhEPO protects small nerve fibers against RTX toxicity by preventing CGRP and SP depletion. Interestingly, some patients can have neuropathic pain or sensory deficit without any change in IENFs density [38]. We hypothesize that functional nerve alterations such as neuropeptide depletion or abnormal nerve conduction could precede nerve degeneration [39]. In this situation, our model might mimic this early stage of sensory neuropathy. Thus, rhEPO could be an efficient treatment in early-stage SFN, that occur in patients exposed to chemotherapy or neurotoxic drugs [40], [41] or in early stage diabetic neuropathy [42], [43]. The mechanism of neuroprotection by rhEPO in our experimental paradigm is still an open issue. A direct effect of erythropoietin on sensory neurons and peripheral nerves is possible, through the binding to the tissue-protective erythropoietin receptor isoform EpoR/β common chain (or CD131) heteromer. Via its receptor, EPO was shown to activate pro-survival signaling through phosphorylation of Janus kinase 2 (JAK2), phosphoinositide 3 kinase (PI3K) and protein kinase B [44]. *In vitro* studies demonstrate that EPO modulate intracellular Ca^2+^ concentration ([Ca^2+^]i), in part via the PI3K pathway, by increasing [Ca^2+^]i in control conditions and decreasing [Ca^2+^]i in pathological conditions [45]. Neuroprotective effect of rhEPO in our study could be mediated by a reduction of [Ca^2+^]i in the excitotoxic condition induced by RTX. Neuroprotective effect of rhEPO could also be central. It was previously shown that peripheral activation of TRPV1 rapidly induces spinal microglia response characterized by increase of iba1 (macrophages/microglia marker) immunoreactivity in spinal dorsal horn [46]. Recent data show that ARA290, a derivative of EPO, exert a strong anti-inflammatory effect by suppression of the spinal microglia response in a mouse model of neuropathic pain induced by spared nerve injury [47]. In our study, rhEPO treament could prevent spinal microglia response induced just after RTX injection, thus facilitating SFN restoration. Further studies will be necessary to clarify this point.

RTX-induced neuropathy was associated with larger stage 2 PU area formation after long ischemic pressure. To exclude microcirculation dysfunctions in cutaneous post-occlusive hyperemia occurring after pressure release (magnet removal), we checked skin microvascular functions by iontophoresis. Ach- and SNP- iontophoresis responses showed that RTX did not alter endothelial or vascular smooth muscle cells, respectively. This analysis allows us to exclude microcirculation dysfunction in the increase of PU development in our sensory neuropathy model induced by RTX. RhEPO prevented the RTX mediated increase of PU areas. RhEPO completely prevented SP/CGRP depletion in TRPV1-expressing DRG neurons of RTX mice. These results suggest that C- and Aδ- nerve fibers impaired by RTX and protected by rhEPO are implicated in skin protection against ischemic pressure injury.

To go further in exploration of the link between neuropeptide depletion and PU development, we treated healthy mice with CGRP and SP antagonists. Healthy mice treated with CGRP antagonist (CGRP 8-37) or NK_1_ antagonist (SR140333) developed a larger lesion area than untreated healthy mice, 24 h after release from a long and occlusive ischemic pressure. Antagonism of SP and CGRP signaling pathways decreased both and independently the skin capacities to protect against long and occlusive pressure. CGRP and SP are the most common and best-studied neuropeptides involved in neurogenic inflammation. “Neurogenic inflammation” refers to inflammatory changes (vasodilatation, plasma extravasation, hypersensitivity) resulting from the release of substances from sensory nerve terminals during injury [48]. We suppose that vascular changes, induced by CGRP (hyperaemia) [49] and SP (plasma extravasation) [50], which occur after pressure release, are essential to protect the skin against pressure-induced ulcer. Both CGRP and SP enhance inflammatory cell infiltration by locally increasing blood flow and stimulating mast cell degranulation [51]. Our data show that depletion of CGRP and SP in cutaneous small nerve fibers lead to an increase of necrosis and a reduced recruitment of inflammatory cells in ulcer tissue. Thus, normal cutaneous neurogenic inflammation seems crucial to protect skin against necrosis extent in PU formation. In addition to their vascular effects, many trophic properties of SP and CGRP have been reported [52]. SP and CGRP stimulate migration and proliferation of keratinocytes and fibroblasts, contribute to neovascularization and thus facilitate wound healing and angiogenesis [10], [53]. Seventy-two hours after pressure release, SP and CGRP depletions in small nerve fibers are associated with reduced cell proliferation and delay in the beginning of skin regeneration processes. Thus, in addition to impairing nociception, alteration of skin nerve fibers by CGRP/SP depletion may impede the normal protective response of the skin to ischemia and the first steps of wound repair. The finding that PU formation is enhanced by SFN, in the absence of microangiopathy is highly reminiscent of what can be found in the human hereditary sensory and autonomic neuropathies [4], [54], [55]. These observations may provide some clues about the pathogenesis of skin lesions in these patients.

General property of rhEPO to improve tissue tolerance against ischemia via its non-hematopoietic effect has been demonstrated in various organs and in particular in the skin [56]. This hypothesis may be excluded, because we found that rhEPO treatment had no protective effect on ischemic injury in untreated Vehicle mice. RhEPO may also have exerted its protective effects through a hematocrit rise, thereby allowing increased tissue oxygenation once pressure had been released. However, both RTX and Vehicle group treated with rhEPO showed an increase of hematocrit, but these mice developed macroscopical and histological lesions similar to those of untreated Vehicle mice. In our model, rhEPO appears to reduce PU formation through its neuroprotective effects only.

In summary, our results strongly suggest that systemic rhEPO pretreatment protects the RTX-mediated peptidergic fibers impairment and thus, prevents PU development. TRPV1-expressing small nerve fibers, which produce CGRP and SP, play a decisive role in protecting the skin from necrosis induced by ischemic pressure. Clinical studies have already shown that systemic or topic EPO treatments had beneficial therapeutic effects on wound healing of chronic skin ulcers [57]. However, systemic EPO would expose to an undesirable increased in hematocrit associated with consequences such as increased risk of hypertension, thrombosis or myocardial infarction [28]. Alternative route of administration, such as topical EPO treatment, could be a solution to avoid systemic EPO side effects [58]. Moreover, the adverse effects of EPO have prompted the discovery of novel derivatives of EPO, devoid of hematopoietic properties, but which conserve tissue protective effects of rhEPO. ARA290, a non- erythropoietic analogue of EPO, showed a significant improvement of neuropathic symptoms in patients with sarcoidosis in a phase II clinical trial [59]. Based on these findings, we believe that rhEPO and its non-erythropoietic analogues could also be used as a preventive method to protect cutaneous nerve fibers and to avoid excessive ulcer formation during situations such as protracted bed-rest or during long surgical procedures in patients who express a SFN.
